# *Eucommia ulmoides* leaf extract alters gut microbiota composition, enhances short‐chain fatty acids production, and ameliorates osteoporosis in the senescence‐accelerated mouse P6 (SAMP6) model

**DOI:** 10.1002/fsn3.1779

**Published:** 2020-07-19

**Authors:** Xin Zhao, Yajing Wang, Zhiying Nie, Lifeng Han, Xinqin Zhong, Xiaohui Yan, Xiumei Gao

**Affiliations:** ^1^ Key Laboratory of Pharmacology of Traditional Chinese Medical Formulae Ministry of Education Tianjin University of Traditional Chinese Medicine Tianjin China; ^2^ Tianjin State Key Laboratory of Modern Chinese Medicine Tianjin University of Traditional Chinese Medicine Tianjin China; ^3^ Tianjin Key Laboratory of Traditional Chinese Medicine Chemistry and Analysis Tianjin University of Traditional Chinese Medicine Tianjin China

**Keywords:** *Eucommia ulmoides* leaf, gut microbiota, osteoporosis, SCFAs

## Abstract

The bark and the leaf of *Eucommia ulmoides* Oliv. content similar bioactive components, but the leaf of this medically important plant is mostly abandoned. In this study, we revealed that the aqueous extract of *E. ulmoides* leaf (EUL) can promote the growth of the probiotic *Lactobacillus bulgaricus* (LB) and inhibit the formation of osteoclast in vitro. This extract was next administrated to senescence‐accelerated mice P6 to evaluate examine its influence on the composition of gut microbiota (GM), short‐chain fatty acids (SCFAs), and osteoporosis (OP). The results showed that supplementation of the EUL aqueous extract to the mouse model: (a) increased bacterial diversity and Firmicutes/Bacteroidetes ratio in the gut, (b) increased SCFAs concentration in the feces and serum, and (c) ameliorated OP based on the results of bone mineral density (BMD), Dual‐energy X‐ray bone scan, and HE staining of distal femur.

## Graphical abstract

The aqueous extract of *E. ulmoides* leaf altered gut microbiota composition, enhanced short‐chain fatty acids production, and ameliorated osteoporosis in the osteoporotic mouse model. This study should promote the future development of EUL as a functional food to treat OP.

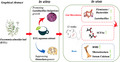

## 1 INTRODUCTION

*Eucommia ulmoides* Oliv., a deciduous tree mainly grown in the south of China, has been used as a Traditional Chinese Medicine (TCM) for more than 3,000 years (Wang, Tang, He, Li, & Wang, 2019). The bark of *E. ulmoides* was used in combination with other herbs to treat hypertension (Greenway, Liu, Yu, & Gupta, 2011; Zhang, Kang, Guo, Zhang, & Ma, 2001). It showed satisfactory clinical efficacy, and safety without obvious adverse reactions (Greenway et al., 2011). It is also used as a tonic and functional food in China, Japan, Korea, and other countries (Yang, Zhang, Fu, & Wu, 2011; Yukihiro, Miyazawa, & Kojima, 2010). Phytochemical and pharmacological studies revealed that bioactive components, including lignans, iridoids, phenolic acids, and flavonoids, are accounting for the antihypertensive, antioxidant, and antiobesity activities of this medicinal herb (He et al., 2014; Yen & Hsieh, 2000). Active chemical components of *E. ulmoides* leaf (EUL) and bark are similar (Yan et al., 2018; Zhang, Wang, Zhang, & Chen, 1996). Compared with the bark, the EUL is richer in resources, but its utilization rate is relatively low (Xing et al., 2019). According to the Pharmacopoeia of China, EUL has been added to the new list of TCM that can be used as medicine homologous food. This would greatly facilitate the utilization of EUL as a functional food.

Osteoporosis (OP), the most prevalent bone disease in the world, is characterized by bone loss, bone tissue microstructure degradation, destruction, bone fragility, and fracture risk (Alejandro & Constantinescu, 2017). It was estimated that half of women and one quarter of men over 50 will experience an OP‐related fracture, and the number of people with OP will increase to 1.5 billion by the year 2050 (Eastell & Lambert, 2002). The surge of OP leads to a heavy burden to the healthcare system. Bone undergoes dynamic remodeling by bone formation and bone resorption (Kini & Nandeesh, 2012). Various antiresorptive agents, such as bisphosphonates, calcitonin, selective estrogen response modulators (SERMs), estrogen, have developed to inhibit bone resorption (Cheng, Wentworth, & Shoback, 2020). Because of the coupling between bone formation and bone resorption, these drugs also reduce bone formation during the treatment. Furthermore, prolonged use of antiresorptive agents, such as bisphosphonates and denosumab, can lead to osteonecrosis of the jaw and atypical femoral fracture (Anastasilakis, Polyzos, & Makras, 2018). These limitations encourage the development of new and safe anti‐OP regimens that can restore skeletal structure and integrity. Extracts from *E. ulmoides* bark have been reported to exert its effects on prevention of estrogen deficiency‐induced bone loss in vivo and in vitro through actions on promoting osteoblasts and inhibiting osteoclasts (Zhang et al., 2009, 2014).

Gut microbiota (GM) is a general term used to denote the complex and dynamic ecology of microorganisms in the gastrointestinal tract. Probiotics (especially *Lactobacillus* species) are also involved in immune regulation, drug/diet metabolism, and synthesis of biologically active substances (Tang & Lu, 2019; Thursby & Juge, 2017). GM‐derived metabolites, such as short‐chain fatty acids (SCFAs), regulate intestinal barrier, mucus, and intestinal functions to maintain intestinal homeostasis, as well as cellular processes (Lin & Zhang, 2017). Growing evidence demonstrates that the host's bone health is closely related to the GM (Sjögren et al., 2012) and SCFAs can enhance bone mineral density (BMD) by reducing osteoclast differentiation and inhibiting bone resorption (Lucas et al., 2018).

In this study, EUL aqueous extract was selected based on its growth promotion effect on *Lactobacillus bulgaricus* (LB) and further evaluated by the inhibitory effect in osteoclast formation. The GM diversity and composition, the SCFAs production, and the anti‐OP effects of EUL aqueous extract were evaluated in senescence‐accelerated mice P6 (SAMP6, P6). With the supports of fecal microbiota composition analyses, SCFAs production, as well as bone microstructure observation, we propose that EUL can be developed as a functional food to treat OP, regulate GM, and increase SCFAs production.

## 2 MATERIALS AND METHODS

### 2.1 Experimental animals

Experimental animals, including 4‐month‐old male SAMP6 (specific pathogen free, SPF level) with OP and 4‐month‐old male anti‐senescence‐accelerated mice R1 (SAMR1, R1; SPF level), were provided by Department of Experimental Animal Science of Peking University Health Science Center (Beijing, China). Mice were raised in the Institute of Radiation Medicine Chinese Academy of Medical Sciences (Tianjin, China), at room temperature (22 ± 2°C), with relative humidity of 58%–65% and light cycle of 12 hr alternation. In this study, ten mice were tested for each group. This study was carried out in strict accordance with the recommendations in the Guidance Suggestions for the Care and Use of Laboratory Animals issued by the Ministry of Science and Technology of China, and approved by the Laboratory Animal Ethics Committee of Tianjin University of Traditional Chinese Medicine (TCM‐LAEC20200022).

### 2.2 Experimental strain and medium

The microorganism *L. bulgaricus* CICC6045 used in this study was purchased from Guangdong Culture Collection Center and preserved at Tianjin University of Traditional Chinese Medicine. *L. bulgaricus* CICC6045 was revived in MRS medium (Solarbio, China) at 37°C, for 24 hr. The cells of the strain were collected by centrifuged at 3,220*g* for 15 min and resuspended in normal saline and then prepared to be microbial agent of 10^8^ CFU/ml for further animal feeding (group LB, *n* = 10). The bacterial solution was administrated by gavage to P6 mice with OP.

### 2.3 Preparation and dose of EUL extracts

*Eucommia ulmoides* leaf was collected in August 2017 from the medicinal botanical garden of Jiangxi Pu Zheng Pharmaceutical Co., Ltd (Jiangxi, China). The EUL was cut in the lab and then extracted with different solvent (volume ratio, 1:8) for twice (1.5 hr each time), respectively, including distilled water, 30% ethanol, 60% ethanol, 75% ethanol, and 90% ethanol. Crude extract was obtained by rotary evaporation distillation at 60°C with speed of 400 ml/hr. Dry extracts were collected at temperature of 60°C under vacuum condition for 48 hr, and the yield of dry extract was calculated to be 33.3%. The extract sample was stored at 4°C. Active constituents of different EUL extract were qualitatively and quantitatively analyzed by HPLC (Figure S1; Wu et al., 2018).

Extract with strong bacteria growth‐promoting properties was used in the following experiments. The low dose of EUL extract (LO, *n* = 10) for treating P6 mice was designed as 1.5 g/kg, and the high group (HI, *n* = 10) was 3.0 g/kg, correspondingly the extract dose was 0.495 g/kg and 0.99 g/kg (Table S1). The above extract was dissolved into water for regularly feeding per mice per day for 12 weeks. The control group of R1 mice (*n* = 10) and the model group of P6 mice (*n* = 10) were given the same volume of pure water. The material was intragastrically administered to SPF mice, and mice were fed to meet the SPF level nutrition needs.

### 2.4 Detection of growth effect of EUL extract on the LB strain

The suitable dilution strain culture was used as plating culture according to previous study (Zhao, Ai, Mao, & Gao, 2019). The MRS medium containing different concentration of EUL extract was used for culturing the LB strain. Dry EUL extract was resolved in sterilized water, passed through a 0.45 µm membrane filter and then added into the sterilized MRS medium. The concentrations of EUL extract were finally adjusted to 200, 100, 50, 10, 5, and 1 mg/ml by serial dilution. The plate with no EUL extract was used as a control. Afterward, 100 μl plating culture was plated on the prepared medium and statically incubated for 24 hr (stationary growth phase reached) at 37°C. Colony numbers were obtained by counting the incubation plates. Three plates were prepared for each condition, and each experiment was repeated twice. The growth value was equal to the colony numbers of strains incubated with EUL extract divided by the colony numbers of strains incubated without EUL extract. Growth promotion activity of EUL extract was presented by a growth value > 1.0, whereas growth inhibition activity by a growth value < 1.0.

### 2.5 In vitro evaluation of EUL extract on osteoclast formation

The bone marrow mononuclear cells of healthy C57BL/6J mice were cultured overnight, and the suspension cells were harvested. The cells were diluted to 3 × 10^5^ cells/ml and added into 96 well plate with 25 ng/ml macrophage colony‐stimulating factor (M‐CSF; 2.5 μl/ml) for 72 hr, to induce osteoclast precursor cells formation. Afterward, EUL extract (0.01 and 0.1 mg/ml of raw material) together with 25 ng/ml M‐CSF (2.5 μl/ml) and 50 ng/ml (5 μl/ml) receptor activator of NF‐κB ligand (RANKL) was added into each well, respectively. By osteoclastic induction for seven days, the tartrate‐resistant acid phosphatase (TRAP) activity in the osteoclast secretion was detected by measuring absorbance at 405 nm, and the pit area of the absorption lacuna was also calculated by bone slice simulation under microscopy (Rico & Villa, 1993). The cells without induction were used as control.

### 2.6 Fecal sample collection, genome DNA extraction, and sequencing

Fresh fecal samples (five of ten mice per group) were collected under sterile condition and immediately frozen at −80°C before euthanasia. Afterward, the genomic DNA was extracted by CTAB/SDS method and the concentration of each DNA sample was quantified to 1 ng/μl. Genome DNA was then sent for sequencing (Novogene, Beijing, China).

The library of 16S rRNA gene V4 region was constructed, qualified, and sequenced on an Illumina HiSeq 250 platform following the procedure in Zhao et al. (2019). Qualified genomic DNA samples were randomly interrupted to about 350 bp by Covaris ultrasonic fragmentation. The whole library was obtained by terminal repair, purification and amplification and then assessed on the Qubit 2.0 Fluorometer (Thermo Scientific) and Agilent Bioanalyzer 2100 system. Afterward, the library was quantified by qPCR with an effective concentration > 3 nM and sequenced on Illumina PE150 platform.

### 2.7 Sequencing data assembly, annotation and analysis

The raw data of 16S rRNA sequencing were analyzed using a standard bioinformatics approach by QIIME software (V1.7.0) according to Zhao et al. (2019). The clean data of metagenome were acquired by Readfq V8 and assembled with scaftigs without N and analyst by SOAPdenovo software (Luo et al., 2012). The ORF was predicted from scaftigs (>=500bp) by MetaGeneMark software (Nielsen et al., 2014). Initial gene catalogues were obtained by removal of redundancy on CD‐HIT (Li & Godzik, 2006). The number of reads and their abundance in each sample were calculated by mapping the clean data to initial gene catalogue using Bowtie2.2.4 (Li et al., 2014). Gene catalogue (unigenes) was acquired by filtering the number of reads <=2 genes and then blasted to the sequences of microorganisms recorded in NR database of NCBI. Annotation information was further confirmed by LCA algorithm of MEGAN software (Huson, Mitra, Ruscheweyh, Weber, & Schuster, 2011).

### 2.8 Analysis of SCFAs production

Blood serum samples (each about 150 μl) and fecal samples (each about 50 mg), after dilution with 50 μl of 15% phosphoric acid, 100 μl of 125 μg/ml of isohexanoic acid (internal standard), and 400 μl ether, were centrifuged at 4°C and 12,400*g* for 10 min, and the supernatant was taken for gas chromatography (Thermo TRACE 1310‐ISQ LT GC/MS, USA) analysis with Agilent hp‐innowax capillary column 30 m × 0.25 mm × 0.25 μm; ion source temperature, 230°C; detector temperature, 250°C; carrier helium, 1.0 ml/min. MS condition was EI source, SIM scanning mode, electron energy 70 eV. Acetic acid, propionic acid, isobutyric acid, butyric acid, isovaleric acid, valeric acid, and caproic acid were detected as standard reference materials.

### 2.9 Bone microarchitecture assessment and microscopic observations

The double‐long bone and muscle tissue of mice were separated in d‐PBS by sterile scissor forceps and then placed in the whole culture of αMEM with 10% FBS and 1% double antibody. Bone microarchitecture was detected by dual‐energy X‐ray bone densitometer with UltraFocus DXA 10*15 Mfg digital radiography system (Hologic Company, USA). The right femur was fixed on the frame of plexiglass box and scanned by faxitron vision with dual‐energy X‐ray absorptiometry. The whole femur was imaged and bone mineral density (BMD) was measured. Femoral specimens were decalcified by EDTA and fixed with 4% polyformaldehyde and then embedded in paraffin when bone tissue became soft. Histological observation of femur was performed by hematoxylin‐eosin (HE) staining, and the number of trabeculae in distal femur was observed by microscope. Serum was collected from eyeballs and stored at room temperature for 2 hr after centrifugation at 800*g* for 15 min at −80°C. Serum calcium concentration and serum phosphorus concentration were detected by kits (Solarbio, China).

## 3 RESULTS

### 3.1 Aqueous extract of EUL promoted the growth of LB

**FIGURE 1:**
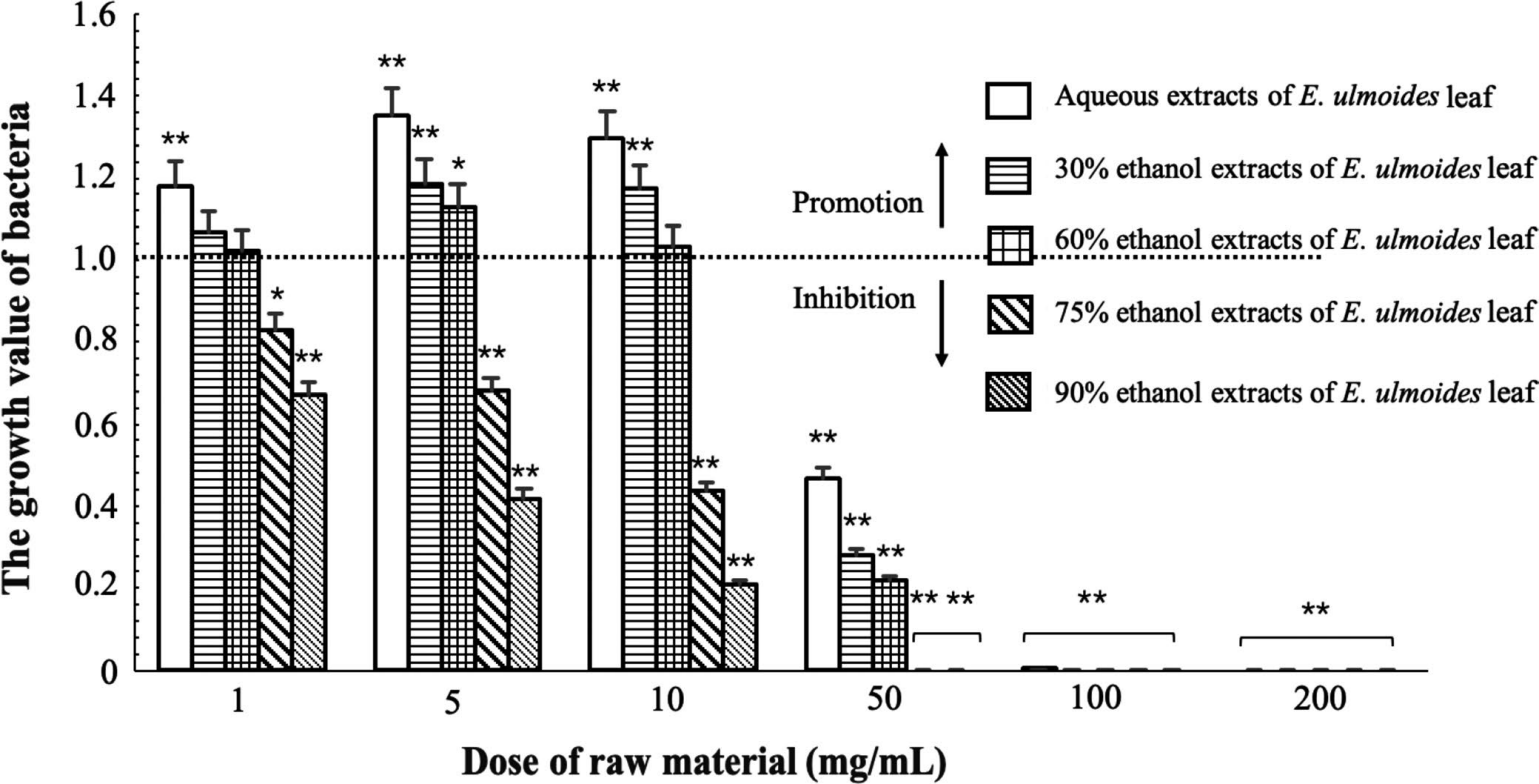
Effects of different *Eucommia ulmoides* leaf extracts on the growth of LB. The growth value equals the colonies of LB supplemented with EUL extract divided by the colonies of LB without EUL extract. A growth value > 1.0 stands for a growth promotion activity of EUL extract, and a growth value < 1.0 stands for a growth inhibition activity of EUL extract (*p < .05, **p < .01)

Various *Lactobacillus* strains, such as *L. reuteri*, *L. bulgaricus*, *L. rhamnosus*, and *L. paracasei*, have been widely investigated for the intervention of aging or OP induced by estrogen deficiency (Britton et al., 2014; Li et al., 2016; Narva, Nevala, Poussa, & Korpela, 2004; Zhao et al., 2019). We firstly investigated the effect of EUL extract on *L. bulgaricus* (LB) growth using the colony‐counting method. The LB culture was adjusted to approximately 2.4 × 10^6^ CFU/ml and used for the assay. The LB colonies in aqueous extract, 30% ethanol extract, and 60% ethanol extract of EUL (1–10 mg/ml) were more than that in the control group (the same inoculum without EUL extract), indicating the growth promotion activity of these three extracts on LB (Figure 1). The number of LB was the highest at the concentration of 5 mg/ml EUL, with a significant growth‐promoting effect (*p* < .01). When the concentration of extracts was higher than 50 mg/ml, they exhibited significant growth inhibition effects on LB (*p* < .01). The number of living LB in 75% ethanol and 90% ethanol extract groups was less than that in control group, indicating their inhibitory effect on the growth of LB (Figure 1). The aqueous extract of EUL had the strongest growth‐promoting effect on LB and was used for the following experiments.

### 3.2 EUL extract suppressed osteoclast formation

**FIGURE 2:**
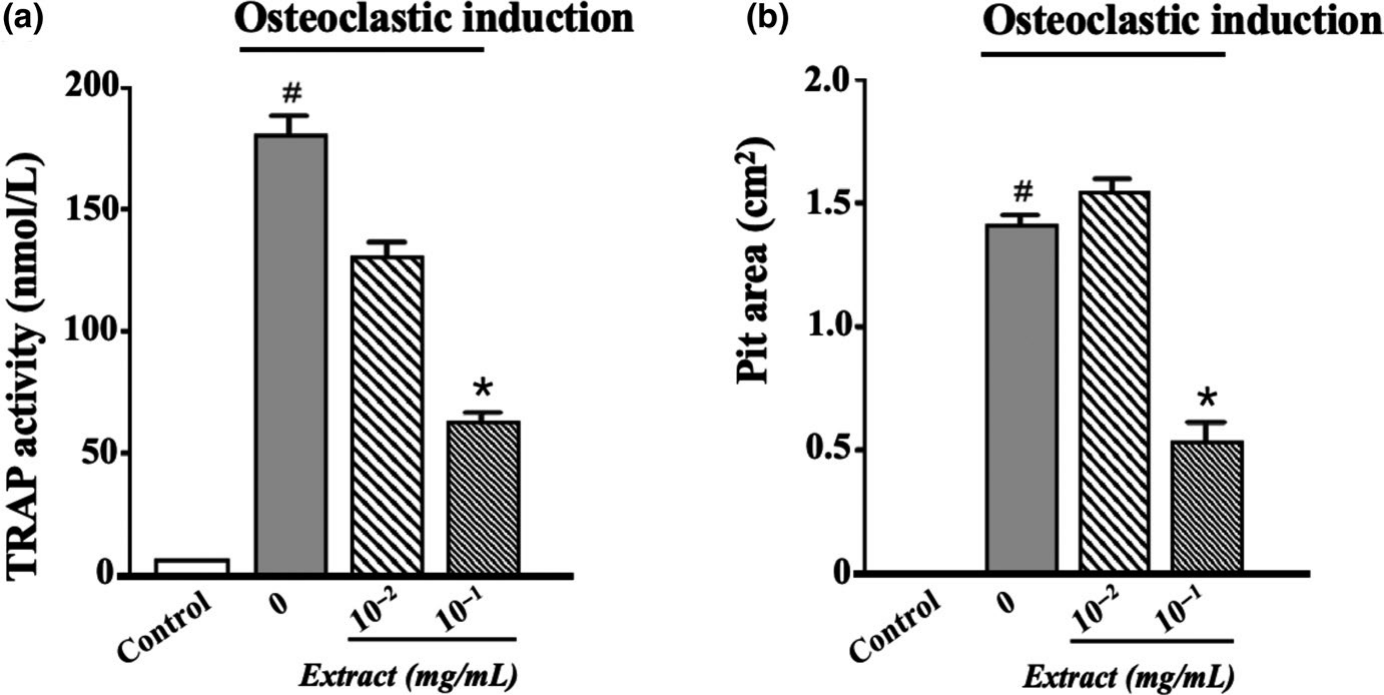
*Eucommia ulmoides* leaf aqueous extract suppressed osteoclastogenesis. (a) TRAP activity test. (b) Pit area calculation of the absorption lacuna by bone slice simulation under microscopy. The bone marrow cell cultures were stimulated with M‐CSF and RANKL and treated with 0.01 mg/ml and 0.1 mg/ml EUL extract, or left untreated (Control) for 7 days (vs. control ^#^ *p* < .05; vs. osteoclastic induction *p < .05)

Under the joint action of M‐CSF and RANKL, bone marrow cells were induced to mature osteoclasts with osteoclastic function. Tartrate‐resistant acid phosphatase (TRAP) activity was often used as an important cytochemical marker of osteoclasts. Compared with uninduced bone marrow cells, the TRAP activity of osteoclast induction group (M‐CSF and RANKL) increased significantly (*p* < .05). When EUL aqueous extract was added to the osteoclast induction group, the TRAP activity was significantly reduced (*p* < .05) in a dose‐dependent manner (Figure 2a). Compared with osteoclast inducer, adding EUL aqueous extract also significantly reduced the pit area of bone lacuna (Figure 2b).

### 3.3 EUL aqueous extract enhanced GM diversity of P6 mice

**FIGURE 3:**
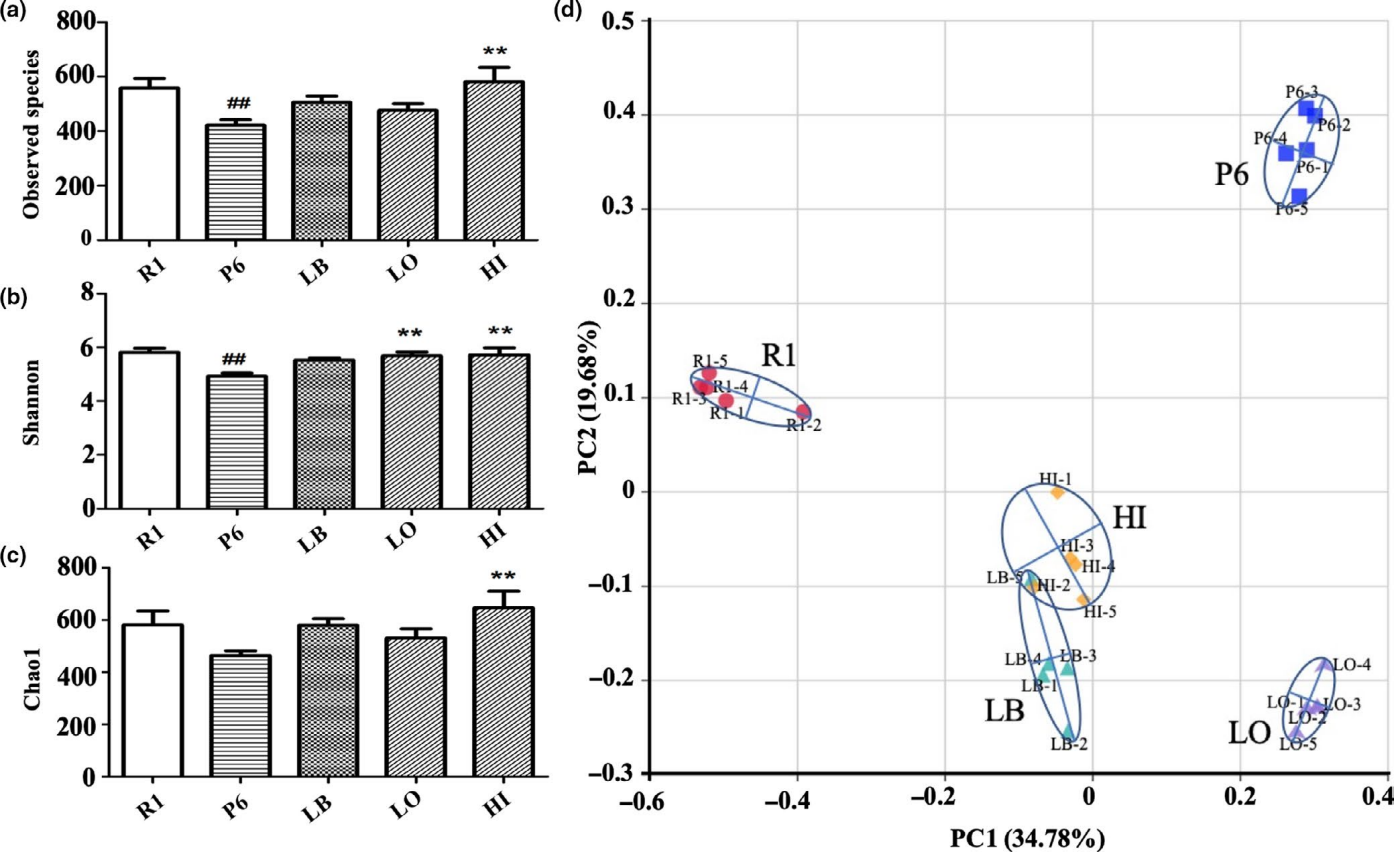
Diversity analysis of GM in the tested mice. Alpha diversity analysis of (a) Observed species; (b) Shannon; (c) Chao1. (vs. R1 group ^#^ *p* < .05, ^##^ *p* < .01, vs. P6 group *p < .05, **p < .01). (d) Weighted UniFrac‐based principal coordinates analysis (PCoA)

The GM composition of each group was determined by 16S rRNA (V4 region) sequencing. An average of 76,780 effective tags was obtained in each sample, with an average of 550 OTU clusters. Alpha diversity analysis, including Shannon (to determine species diversity) and Observed species (to observe the number of species), was used to describe the complexity and species richness of bacteria in different samples (Figure 3a–c). Compared with the R1 group, the Observed species index was significantly lower in the P6 group (*p* < .01). By treatment with LB and EUL extract (LO and HI), the Observed species index in P6 mice was significantly increased (*p* < .01). The same trend was also detected for the Shannon index in the P6 mice. The bacterial diversity of mice with high dose of EUL aqueous extract increased as assessed by Chao1 index (*p* < .01, vs. P6), while no significant difference was noticed between R1 and P6 group. Weighted UniFrac‐based principal coordinates analysis (PCoA) indicated the obvious difference between the P6 group and the R1 group after the 12‐week administration, and the LB and EUL extract groups located between the R1 and P6 samples (Figure 3d).

### 3.4 EUL aqueous extract increased the abundance of Firmicutes and other SCFA‐producing bacteria in P6 mice

**FIGURE 4:**
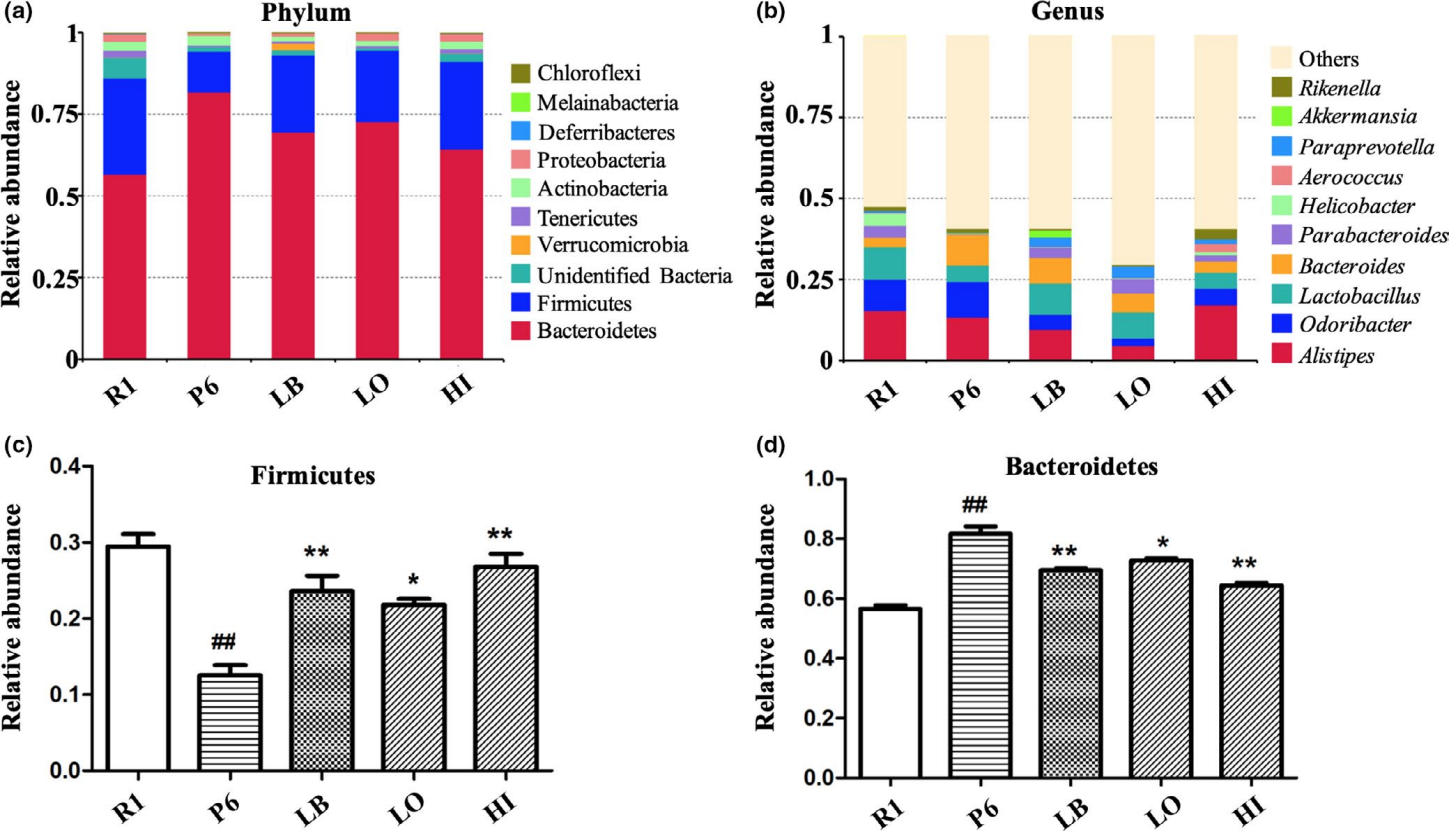
The relative abundance of bacterial communities of different groups at the phylum level (a) and the genus level (b). Statistical comparison of the relative abundance of Firmicutes (c) and Bacteroidetes in the tested mice. (vs. R1 group ^#^ *p* < .05, ^##^ *p* < .01, vs. P6 group *p < .05, **p < .01)

Metagenomic sequencing of the fecal samples was performed to evaluate the impact of EUL extract on the GM composition in the P6 mice. At the phylum level, Bacteroidetes, Firmicutes, Actinobacteria, unidentified Bacteria, Verrucomicrobia, Tenericutes, and Proteobacteria are the most abundant bacteria in mice GM. Compared with R1 mice, P6 mice possessed more Bacteroidetes, but less Firmicutes, Verrucomicrobia, Tenericutes, and Proteobacteria. When EUL extract was administrated in the P6 mice (HI and LO group), the GM composition was altered toward that of the group R1, as can be seen in the significantly increased abundance of Firmicutes and decreased abundance of Bacteroidetes (*p* < .01, vs. P6; Figure 4a,c,d). At the genus level, the most abundant bacteria in mice GM included *Alistipes*, *Odoribacter*, *Lactobacillus*, *Bacteroides*, *Parabacteroides*, *Helicobacter*, *Aerococcus*, *Paraprevotella*, *Akkermansia*, and *Rikenella*. Compared with R1 mice, P6 mice featured more *Bacteroides*, *Odoribacter* and *Rikenella*, but less *Alistipes*, *Lactobacillus*, *Parabacteroides*, *Helicobacter*, *Aerococcus*, *Paraprevotella*, and *Akkermansia*. When LB, EUL extracts (HI or LO) were respectively supplemented to the P6 mice, abundance of *Lactobacillus*, *Parabacteroides*, *Aerococcus*, *Akkermansia*, and *Paraprevotella* wasere increased and that of *Bacteroides* and *Odoribacter* decreased. The abundance of *Lactobacillus* was the highest in the R1 group, followed by the LB and the LO group (Figure 4b). In addition, the abundance of SCFAs producers, including *Roseburia*, *Blautia*, and *Faecalibacterium* was higher in both the LB and the HI group than that in the P6 group.

### 3.5 EUL aqueous extract enhanced SCFAs production in both the feces and the serum of P6 mice

**FIGURE 5:**
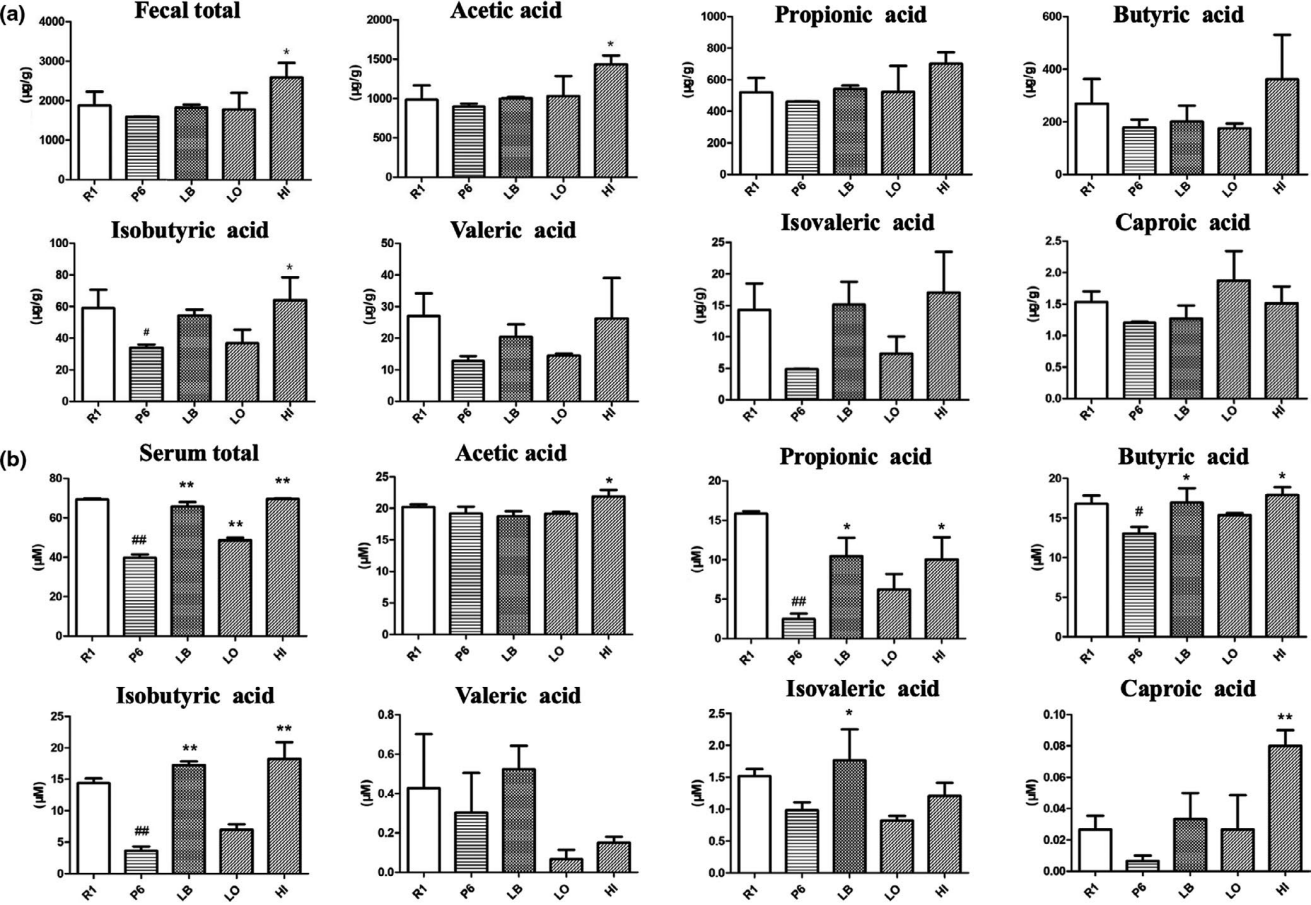
Short‐chain fatty acids concentration in the fecal (a) and the serum (b) samples of different groups (vs. R1 group ^#^ *p* < .05, ^##^ *p* < .01, vs. P6 group *p < .05, **p < .01)

Concentration of SCFAs, such as acetic acid, propionic acid, isobutyric acid, butyric acid, isovaleric acid, valeric acid, and caproic acid, in both fecal and serum samples of the tested mice was analyzed by GC‐MS. As shown in Figure 5a, total concentration of SCFAs in the fecal samples was lower in group P6 than in group R1. When LB and EUL aqueous extracts were supplemented to P6 mice, respectively, the total amount of SCFAs increased. EUL extract high‐dose administration (HI) remarkably increased the production of acetic acid and isobutyric acid (*p* < .05) analyzed with respect to P6. There was also an increase (vs. P6) of all tested SCFAs in the LB group. Through GC‐MS analysis of the serum samples, we found that the total concentration of SCFAs was significantly lower in group P6 (*p* < .01) compared with R1, and significantly increased in all the supplementation groups (Figure 5b). Concentration of propionic acid (*p* < .01), butyric acid (*p* < .05), and isobutyric acid (*p* < .01) was lower in the P6 group than the R1 group. Serum concentration of these three SCFAs was increased by supplementation with LB and high dose of EUL extract, except valeric acid whose serum content was reduced upon EUL extract administration (Figure 5b). Supplementation of low dose of EUL extract enhanced production of most the SCFAs except valeric acid, but with no statistically significant difference.

### 3.6 EUL aqueous extract ameliorated osteoporosis in the OP mice

**FIGURE 6:**
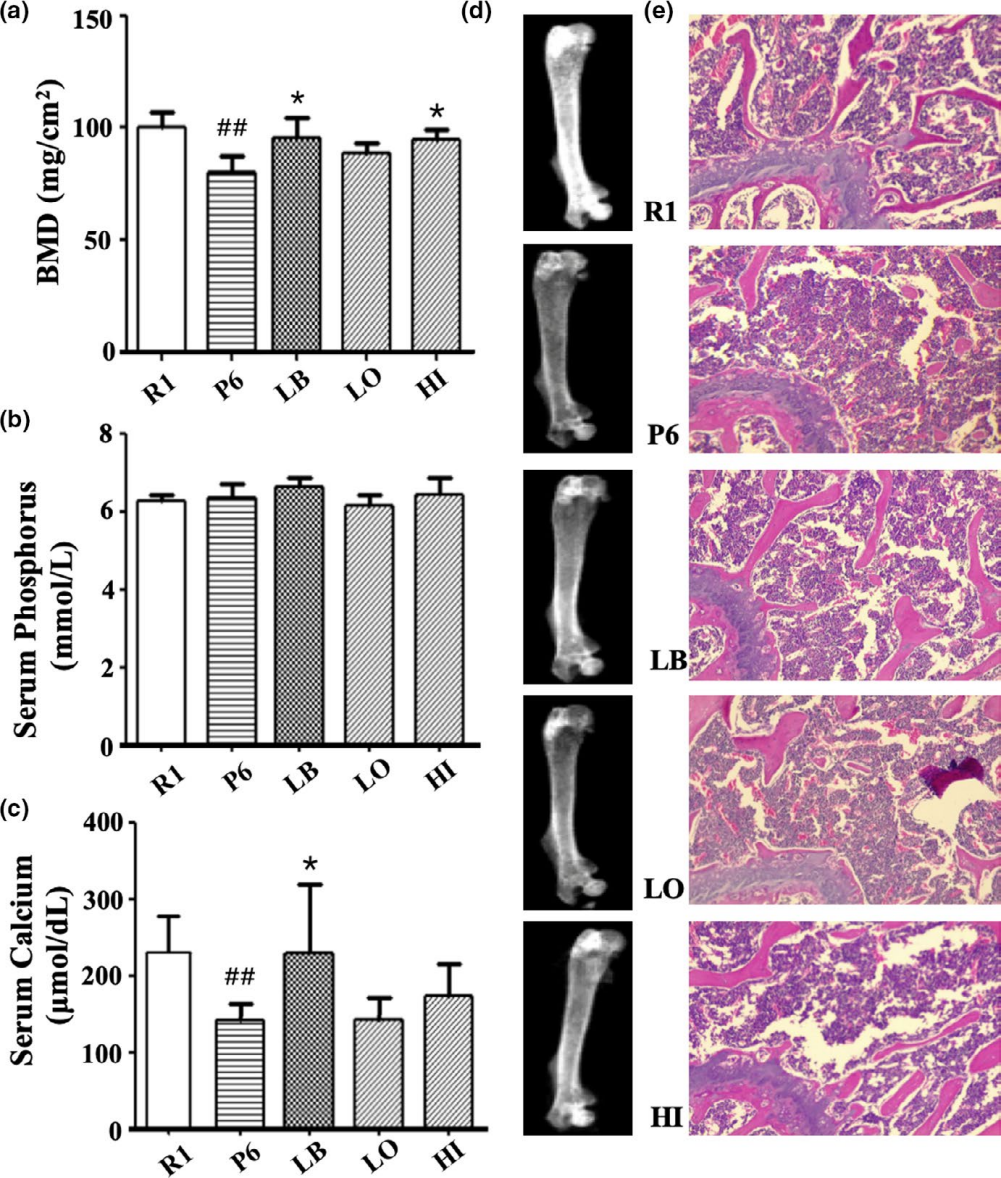
Effects of LB and *Eucommia ulmoides* leaf aqueous extract on (a) the BMD in the femur, (b) the serum calcium and (c) the serum phosphorus levels of mice. (vs. R1 group ^#^ *p* < .05, ^##^ *p* < .01, vs. P6 group *p < .05, **p < .01). (d) Dual‐energy X‐ray bone scan of mice femur. (e) HE staining of distal femur in the tested mice (×100)

Bone mineral density is the gold index in the diagnosis of OP. Compared with the control group R1, the BMD of femur in P6 model group decreased significantly (*p* < .05). When high dose of EUL extract (HI) and *L. bulgaricus* bacteria (LB) were respectively administrated to the P6 mice, the BMD increased significantly (*p* < .05) and reached to the same level as the R1 mice. In the EUL extract low‐dose group (LO), the BMD was also slightly increased (*p* < .05, Figure 6a). There was no significant difference in the serum phosphorus levels among all tested mice (Figure 6b). Compared with the R1 group, the serum calcium content of P6 model mice was significantly lower (*p* < .01). The serum calcium content of P6 mice was significantly increased in the LB group (*p* < .05) and slightly increased by in the LO and HI groups (Figure 6c). The dual‐energy X‐ray graph showed that bone condition in P6 mice could be improved by feeding with LB and high dose of EUL extract (Figure 6d). HE staining results of mice femoral tissue sections were shown in Figure 6e. Compared with the R1 group, the P6 mice featured sparse distal femoral trabeculae, reduced trabeculae, and larger trabecular space. The number of bone trabeculae of P6 mice increased upon treatment with LB and aqueous extract of EUL. Moreover, the number of bone trabeculae of HI group was larger than that of LB group (Figure 6e). The results showed that EUL extract could inhibit bone loss in mice.

## 4 DISCUSSION

The stem and bark of *E. ulmoides* have been used as a folk medicine in China, Korea, and Japan for hundreds of years, to treat lumbago induced by kidney deficiency and to increase human longevity (Wang et al., 2019). It is now listed in the Pharmacopoeia of China as a medicine to cure hypertension, impotence, lumbago, and ischialgia. The extensive peeling of the stem and bark of *E. ulmoides* endangers the supply of this valuable plant. At the same time, EUL, whose harvest has minimal effect on the growth of this deciduous tree, was mostly abandoned and wasted. Recent studies have revealed that the EUL content similar bioactive components as the bark, including aucubin, geniposidic acid, chlorogenic acid, and rutin (Zhang et al., 1996). In this study, we confirmed that these quality control markers for *E. ulmoides* were readily extracted from the leaf by simple chopping and then soaking in water. The aqueous extract of EUL is recently used as a functional beverage in the treatment of hypertension and hypercholesterolemia (Yukihiro et al., 2010; Zhang et al., 2001). In the latest Pharmacopoeia of China (2015), EUL has been added to the new list of TCM as a medicine food homologous product. This will greatly encourage the utilization of EUL.

Besides the previously reported blood pressure lowering, antioxidation, and immunity enhancing properties, in this study, we discovered that the aqueous extract of EUL altered the bacterial composition of the GM, as well as inhibited the formation of osteoclast. The osteoporotic P6 mice had less GM diversity compared with the health R1 mice, and the R1 mice featured a higher Firmicutes/Bacteroidetes ratio than the P6 mice. *Lactobacillus* is the most studied genus in the Firmicutes phylum, and many metabolites of *Lactobacilli* can inhibit the growth of pathogens and ameliorate GM disorder (Murphy et al., 2013; Wang et al., 2014). Our in vitro experiments revealed the growth promotion activity of aqueous extract of EUL on LB. Administration of the aqueous extract in P6 mice significantly increased the abundance of Firmicutes and decreased the abundance of *Bacteroides*, leading to much higher Firmicutes/ Bacteroidetes ratios in the osteoporotic mice. Compared with the R1 health mice, the P6 mice displayed much less strain diversity, suggesting a fundamental correlation between bone homeostasis and strain diversity in the GM. Administration of aqueous extract of EUL significantly enhanced the strain diversity in the gut of P6 mice, as assessed by the Observed species and Shannon index. The strain composition of mice feed with high dose of EUL extract was similar to that of *Lactobacillus*‐fed mice, suggesting that EUL aqueous extract may exert its anti‐OP activity via a similar mechanism as *Lactobacillus*.

Short‐chain fatty acids are important molecular signals between the microbiota and host, or key mediators of host cellular metabolism. They are generally derived from GM‐dependent fermentation of dietary fibers, then absorbed, and transported via portal circulation to regulate signals through immune modulation and host metabolism (Koh, De Vadder, Kovatcheva‐Datchary, & Bäckhed, 2016). Propionic acid and butyric acid were previously reported as regulators of osteoclast metabolism and bone homeostasis (Lucas et al., 2018). These SCFAs are proposed to promote bone health by regulating the RunX, osteoprotegerin, and nuclear factor κB ligand signaling pathways (Katono et al., 2008; Lee et al., 2006; Rahman et al., 2003). In this study, the abundance of well‐known SCFAs producers, such as *Roseburia, Blautia,* and *Faecalibacterium,* was increased in the R1 mice and P6 mice treated with EUL extract or LB. This result is consistent with the higher SCFAs concentrations in the fecal and serum samples of *E. ulmoides* supplemented P6 mice.

Finally, we evaluated the effect of EUL extract on the bone metabolism in mice by BMD determination, dual‐energy X‐ray graph, and HE staining. Compared with the R1 mice, BMD of the 12‐week P6 mice reduced by 20% and the number of trabeculae decreased obviously. Supplementation of LB and aqueous extract of EHL to the P6 mice enhanced the BMD and the trabeculae, to almost the same levels as the R1 mice. These results undoubtedly supported the anti‐OP activity of EUL aqueous extract, but the mechanism behind this activity remains unknown.

## 5 CONCLUSIONS

To conclude, in this study, we performed preliminary evaluation of aqueous extract of EUL in OP treatment. The in vitro experiments revealed that the aqueous extract of EUL promotes the growth of *Lactobacillus* strain and inhibits the formation of osteoclast; Our in vivo study using the senescence‐accelerated mouse P6 model confirmed the GM modulation, SCFAs production enhancing, and anti‐OP activity of aqueous extract of EUL. Our results provide fundamental basis for further preclinical studies and the eventual development of EUL as a functional food to treat OP.

## CONFLICT OF INTEREST

There are no conflicts of interest to declare.

## ETHICAL APPROVAL

This study was carried out in strict accordance with the recommendations in the Guidance Suggestions for the Care and Use of Laboratory Animals issued by the Ministry of Science and Technology of China. The protocols were approved by the Laboratory Animal Ethics Committee of Tianjin University of Traditional Chinese Medicine (Permit Number: TCM‐LAEC20200022).

## Supporting information

Figure S1

Table S1
