# Supplementary material for: Eucommia ulmoides leaf extract alters gut microbiota composition, enhances short‐chain fatty acids production, and ameliorates osteoporosis in the senescence‐accelerated mouse P6 (SAMP6) model
Source: Food Sci Nutr. 2020 Jul 19;8(9):4897–906. doi: 10.1002/fsn3.1779 (PMC7500782; doi:10.1002/fsn3.1779)
Supplement: Supplementary file 1 — Figure S1 [file FSN3-8-4897-s001.docx]

**Figure S1** Detection of active components in extracts of *E. ulmoides* leaf by HPLC. HPLC of mixed reference solution and *E. ulmoides* leaf extract sample solution. (a), (c), (e), (g), (i) and (k) were mixed reference solution, extracts of *E. ulmoides* leaves in aqueous solution, 30% ethanol, 60% ethanol, 75% ethanol and 90% ethanol extract samples at 208 nm, respectively. (I) was aucubin. (b), (d), (f), (h), (j) and (l) were mixed reference solution, extracts of *E. ulmoides* leaves in aqueous solution, 30% ethanol, 60% ethanol, 75% ethanol and 90% ethanol extract samples at 238 nm, respectively. (II), (III), (IV) were geniposidic acid, chlorogenic acid and rutin. Chromatographic column: Agilent 20RBAX Eclipse Plus C18 (250 mm *4.6 mm, 5 micron); mobile phase: acetonitrile A-0.05% phosphoric acid; B-gradient elution (0-15 min, 4% A 6% A; 15-19 min, 6% A 12% A; 19-34 min, 12% A; 34-55 min, 12% A 20% A); detection wavelength: 208 nm and 238 nm; column temperature: 25 C; flow rate: 1 mL/min; injection volume of 10 *μ*L. The content of active ingredients was calculated by detecting the peak area. Active components content in extracts of *E. ulmoides* leaf (%) were listed in the following Table.

**Table Active components in extracts of *E. ulmoides* leaf (%)**

| Extraction solvent | Aucubin | Geniposidic acid | Chlorogenic acid | Rutin |
| --- | --- | --- | --- | --- |
| Aqueous | 1.223 | 0.689 | 1.166 | 0.059 |
| 30% ethanol | 0.722 | 0.615 | 1.668 | 0.083 |
| 60% ethanol | 0.533 | 0.458 | 1.759 | 0.093 |
| 75% ethanol | 0.378 | 0.433 | 1.726 | 0.085 |
| 90% ethanol | 0.257 | 0.336 | 0.811 | 0.053 |
