# Supplementary material for: Eucommia ulmoides leaf extract alters gut microbiota composition, enhances short‐chain fatty acids production, and ameliorates osteoporosis in the senescence‐accelerated mouse P6 (SAMP6) model
Source: Food Sci Nutr. 2020 Jul 19;8(9):4897–906. doi: 10.1002/fsn3.1779 (PMC7500782; doi:10.1002/fsn3.1779)
Supplement: Supplementary file 2 — Table S1 [file FSN3-8-4897-s002.docx]

**Table S1. Dose of *E. ulmoides* leaf extract (LO and HI) and *L. bulgaricus* (LB) administrated to the P6 mouse model**

| Group | Dose of raw material |
| --- | --- |
| Control (SAMR1, R1) | — |
| Model (SAMP6, P6) | — |
| *L. bulgaricus* (*Lactobacillus*, LB) | 10^8^ CFU/mL |
| *E. ulmoides* Low dose (low, LO) | 1.5 g/kg |
| *E. ulmoides* High dose (high, HI) | 3 g/kg |
